# From Mother to Baby: The Role of Human Milk Vitamins in Infant Body Development and Breast Milk Jaundice—An Observational Study

**DOI:** 10.1002/fsn3.70922

**Published:** 2025-09-15

**Authors:** Yuanyuan Zhang, Xuerong Zhang, Zhenrong Xie, Jingjing Xiong, Meng Li, Zhanhua Li, Yongkun Huang

**Affiliations:** ^1^ Department of Pediatrics The First Affiliated Hospital of Kunming Medical University Kunming China; ^2^ Department of Scientific Research and Education The First Affiliated Hospital of Kunming Medical University Kunming China; ^3^ Clinical Research Center The First Affiliated Hospital of Kunming Medical University Kunming China; ^4^ Yunnan Provincial Key Laboratory of Laboratory Medicine Kunming China

**Keywords:** breastfeeding, breast milk jaundice, human milk, infant growth, vitamins

## Abstract

The vitamins in infants who are exclusively breastfed before 6 months of age mainly come from breastfeeding. Whether or not dynamic vitamin concentrations in human milk were affected by maternal factors and their roles in infant growth and diseases has yet to be determined. Using data from a tertiary hospital for 46 couples of fit mothers and infants aged 1 to 3 months in China, we collected mothers' human milk as samples for UPLC‐MS/MS sequencing evaluation of vitamins. Multiple regression and binomial logistic regression were used in analyses that controlled for confounding. The content of vitamin B1 was significantly higher at 105 to 119 days postpartum than it was at 30 to 44 days after delivery (*p* < 0.05). Additionally, vitamin C was related to infant weight‐for‐age (*β* = 0.05, SE = 0.02, *p* = 0.02). Vitamins C (*β* = 0.00015, SE = 0.00006, *p* = 0.01) and B7 (*β* = 0.00048, SE = 0.00023, *p* = 0.04) were related to infant BMI. After adjustment for sample collection times, vitamins A and B1, the odds ratio for vitamin K in the BMJ group was 13.93 (95% CI, 0.93 to 208.48, *p* = 0.05). Overall, the vitamin B1 contents from human milk change in different stages of lactation. A longer duration of fully breastfeeding with vitamins B7 and C provides better developmental nutrients to infants aged 1 to 3 months. According to our study, vitamin K concentration seems to be increased in the human milk of mothers whose infants had BMJ.

**Trial Registration:** This trial was registered on 22/12/2023 as ChiCTR2300078973

## Graphical abstract

Based on previous studies, this study was the first to interpret the effect of maternal status on variant vitamin secretion in human milk during 1 to 3 months postpartum. The study explored the relationship between the content of vitamins in human milk and the infants with breast milk jaundice who were exclusively breastfed. The research provides new evidence regarding the association between infant growth and human milk vitamins and lays the groundwork for the development of infant's exceptional medical food and infant formula milk.

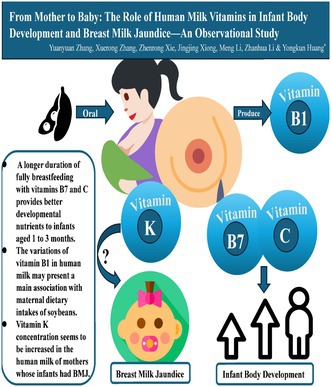

## 1 Introduction

Vitamins, termed as micronutrients, are crucial to multiple biological processes for a rapidly growing infant and need to be replenished with their main nutrition from foods as they are limited stores at birth and cannot be synthesized *de novo* by the human body (Leaf and Lansdowne 2014). Exclusive breastfeeding is recognized as the optimal standard because it plays an undeniable role in the initial 6 months of life nutrition (Bravi et al. 2021). By 4–6 weeks postpartum, the fully mature stage is reached, after which the human milk metabolome remains relatively constant (Kim and Yi 2020; Spevacek et al. 2015). Nevertheless, previous publications have documented that human milk vitamins were subject to change with the progress of lactation, within circadian variation, and, for some, by geographical origin, parity, vitamin supplementation, nutritional status, and diet regime (Dror and Allen 2018; Hampel et al. 2017; Qiao et al. 2022; Redeuil et al. 2021; Ureta‐Velasco et al. 2023; Xue et al. 2017). The impact of main maternal status on vitamin level in human milk and subsequent availability to infants aged 1 to 3 months is poorly understood.

The real breast milk jaundice (BMJ) refers to late‐onset breast milk jaundice, which results in the potential dangers of lipid‐soluble unconjugated bilirubin passing the immature blood–brain barrier (Pattison et al. 2019). It may develop as the result of liver immaturity, the composition of bile acid and fatty acid (Gao et al. 2023; Yang et al. 2020), strong activity of β‐glucuronidase, high levels of epidermal growth factor (Guo et al. 2022), increased IL‐1β (Apaydin et al. 2012), poor intestinal microecological environment (Duan et al. 2021, 2018; Guo et al. 2023; Huang et al. 2023; Li et al. 2020; Zhou et al. 2019), premature birth, and genetic factors (Prameela 2019). Due to the uncertain pathogenesis of BMJ, we currently lack an accurate laboratory testing method to diagnose it. Furthermore, few reports provide data on the relative vitamin factors needed to develop infant physique growth (Reyes et al. 2024), to the best of our knowledge, and no data are available on breast milk jaundice. Therefore, the primary objective of this study was to explore whether the human milk vitamins during 1 to 3 months postpartum could promote body growth and prevent risk factors in BMJ infants who were exclusively breastfed. Another objective was to evaluate whether the changes in dynamic vitamin amounts secreted in human milk over time differed based on maternal factors.

## 2 Methods

### 2.1 Participants

This observational study was conducted from September 2020 to March 2021 in a tertiary hospital in China. The trial recruited 46 couples of fit mothers and infants. The inclusion criteria for all infants were as follows:

1. Age between 1 and 3 months.
2. Were exclusively breastfed in accordance with the recommended daily intakes to ensure adequate nutrition.
3. Term infants of average birth weight and length.
4. Not on additional vitamins other than 1 mg intramuscular of vitamin K as soon as possible after birth and a vitamin D recommended dose of 400 IU per day.
5. Among them, 12 infants with BMJ met the BMJ diagnostic criteria, which included the following:
  - Jaundice appeared between Days 7 and 10 postpartum and continued to rise well beyond the physiological range, with a peak in bilirubin typically occurring 2–3 weeks later and lasting 4–6 weeks or even 2–3 months before fading.
  - After the cessation of human milk for 1–3 days, jaundice significantly subsided, and bilirubin levels rapidly decreased by 30% to 50%.

The exclusion criteria are as follows:

1. Pathological jaundice included infectious, autoimmune hemolysis, cholestasis, glucose‐6‐phosphate dehydrogenase deficiency, hypoglycemia, polycythemia, hypothermia, scalp hematoma, hypothyroidism, intracranial hemorrhage, and perinatal asphyxia.
2. Dystrophy, protein deficiency, vitamin D deficiency, trace element deficiency, malabsorption syndrome, rickets, iron deficiency anemia, persistent diarrhea, chronic enteritis, dysentery, congenital digestive tract malformation, tuberculosis, measles, recurrent respiratory infections, congenital heart disease, immunodeficiency disease, and genetic metabolic disorders.
3. Use of phototherapy, liver enzyme inducers, medicinal charcoal, agar, montmorillonite powder, and probiotics for treatment.
4. Poor feeding and abnormal urine and stool, as well as improper weight gain.

The corresponding mothers met the following criteria:

1. Age ≥ 18 years.
2. Excellent physical and mental health during pregnancy and breastfeeding.
3. Well‐nourished.
4. Healthy breast status.
5. Not on any vitamin supplements during lactation.

The exclusion criteria are as follows:

1. Absence of gestational diabetes mellitus, gestational hypertension, and hypothyroidism.
2. Absence of anemia and malnutrition.
3. Absence of neuropsychiatric disorders.
4. Lack of medical family history.
5. Absence of poor habits.

### 2.2 Data Collection

Information on mother‐infant pair characteristics, gestation period, and breastfeeding situation, as well as other relevant environmental factors, was self‐reported by mothers in a face‐to‐face interview using questionnaires. Additionally, anthropometric parameters such as length and weight were measured by the dedicated researchers. BMI was calculated as body weight divided by length squared (kg/m^2^). Infant weight/length for age was calculated as the current weight/length minus the average weight/length and then divided by a standard deviation of 10 times, and finally 50 was added (NFS 2006).

### 2.3 Milk Sampling

From 9 a.m. to 11 a.m., all mature milk samples were collected manually with sterile gloves from the mother's breast before feeding the infant, and the milks (30–44, 45–59, 60–74, 75–89, 90–104, and 105–119 days *postpartum*, respectively) were aliquoted into 2 mL sterile containers and stored at −80°C.

### 2.4 Measures

#### 2.4.1 Water‐Soluble Vitamins (B1, B2, B3, B5, B6, B7, B9, B12, and C) Quantification

One aliquot of 1 mL from each thawed human milk sample was taken and centrifuged at 3000 rpm for 15 min at 4°C (Centrifuge 5804R, Eppendorf, Germany) to remove fat. After centrifugation and degreasing, the supernatants were extracted with 60 μL in a 1.5 mL centrifuge tube. Then 60 μL of stable isotopically labeled internal standards (Supporting Information) were added to defatted human milk for deproteinization. After shaking and mixing the liquids well for 5 min, the samples were centrifuged at 12000 rpm for 10 min, and then 70 μL was transferred into 96 holes of the sample plate for detection. The preparation of the standards as well as the controls is consistent with the milk samples. Finally, the standards, controls, and samples were analyzed by ultra‐performance liquid chromatography combined with tandem mass spectrometry (UPLC‐MS/MS) performed using a Shimadzu LC‐AD ultrafast liquid chromatography system (Shimadzu, Tokyo, Japan) and an API 4500 triple quadrupole mass spectrometer (SCIEX, Framingham, MA, USA) in the positive ion mode. The chromatographic separation was carried out with a Waters Acquity HSS T3 column (HSS T3, 2.1 × 100 mm i.d., 3 μm). The column temperature was set to 40°C, with a mobile phase (Supporting Information) at a flow rate of 0.5 mL/min. The gradient process was: 0 min, 99% A; 1 min, 99% A; 1.1–1.9 min, 99% A; 2–3.4 min, 2% A; 3.5–4.4 min, 2% A; 4.5–4.9 min, 99% A; 5 min, stop.

The MS conditions for detection of the chromatographic separation were as follows: the ion source temperature was 450°C, and the spray voltage was 5500 V. To demonstrate method reliability, quality control (QC) samples, serving as internal reference materials, were analyzed periodically at predetermined intervals throughout the study. This generated QC curves for ongoing performance monitoring. The method exhibited satisfactory precision, with coefficients of variation (CV) for both repeatability and intermediate reproducibility below 13%. The *R* ^2^ of the standard curve for all standards was more than 0.9900. The limit of detection (LOD) for vitamin B1, vitamin B2, vitamin B3, vitamin B5, vitamin B6, vitamin B7, vitamin B9, vitamin B12, and vitamin C were 0.2, 0.2, 1.563, 1.563, 0.3, 0.156, 0.781, 0.156, and 0.391 (ng/mL), respectively. Recovery rates of vitamin B1, vitamin B2, vitamin B3, vitamin B5, vitamin B6, vitamin B7, vitamin B9, vitamin B12, and vitamin C were 85.53%–95.55%, 88.23%–106.44%, 89.14%–93.74%, 91.06%–97.16%, 95.16%–103.87%, 91.49%–102.98%, 87.20%–97.17%, 99.88%–110.08%, and 87.33%–90.02%, respectively. No crosstalk was observed between internal standards and analytes during scheduled multiple reaction monitoring (MRM) acquisition.

#### 2.4.2 Lipid‐Soluble Vitamins (A, E, and K) Quantification

1 mL of each human milk sample was left at 4°C and centrifuged afterward at 3000 rpm for 15 min (Centrifuge 5804R, Eppendorf, Germany). The upper liquid was gathered with 300 μL into 96 holes in the sample plate, and protein precipitation was performed with the addition of 200 μL of stable isotopically labeled internal standards (Supporting Information). The above liquid was fetched out of 400 μL and pressed through 96 holes of the filter plate. After letting the mixture stand for 5 to 10 min, we added 750 μL of extract solution (Supporting Information) containing n‐hexane and collected the eluent into 96 holes of the collection plate with a positive pressure device. The samples were dried under nitrogen (2 mL/min flow, about 5 min) after repeating this step once. All the human milk samples were added to a 100‐μL methanol aqueous solution containing formic acid as well as ammonium formate and were further shaken vigorously (vortex, 2 min). Samples were then transferred into 96 holes of the inlet plate and submitted for analysis. The processing of standards as well as controls is the same as that of milk samples. After extraction, nitrogen blowing, and redissolving, the samples were tested by ultra‐performance liquid chromatography–tandem mass spectrometry (UPLC‐MS/MS) with a Waters Acquity HSS T3 column (HSS T3, 2.1 × 50 mm i.d., 3 μm) being used for separation. Liquid chromatography (LC) was performed using a Shimadzu LC‐AD ultrafast liquid chromatography system (Shimadzu, Tokyo, Japan). The column temperature was controlled at 40°C. Mobile Phase A and Mobile Phase B served as mobile phases (Supporting Information) at a flow rate of 0.5 mL/min. The gradient process was: 0 min, 20% A; 0.5 min, 20% A; 0.6–1.9 min, 20% A; 2–4.9 min, 2% A; 5–5.9 min, 2% A; 6–6.9 min, 20% A; 7 min, stop. An API 4500 triple quadrupole mass spectrometer (SCIEX, Framingham, MA, USA) was used for detection of the chromatographic separation. The MS performing conditions were as follows: The ion source temperature was 450°C, and the spray voltage was 5500 V. Quality control (QC) samples (internal reference materials) were analyzed periodically in duplicate during runs to monitor performance. Coefficients of variation (CV) for repeatability and intermediate reproducibility were < 13%. Calibration curves for all analytes achieved *R* ^2^ > 0.9500. Limits of detection (LOD) were vitamin A: 12.5 ng/mL; vitamin E: 0.1 ng/mL; vitamin K: 0.094 ng/mL. Recovery rates of vitamin A, vitamin E, and vitamin K were 91.38% to 92.87%, 85.52% to 91.58%, and 92.17% to 109.89%, respectively. Recoveries ranged from 85.52% to 109.89%, with no MRM cross‐talking observed.

### 2.5 Statistical Analysis

Statistical analyses were done with SPSS (ver. 25.0) and R software (ver. 2.15.3). Normally distributed data were presented as means and standard deviations (SD). Data with a skewed distribution were presented as medians with the interquartile range. The categorical data were expressed as a percentage. The Mann–Whitney *U* test and Kruskal‐Wallis *H* test (with Dunn's post hoc test) were used for comparisons of discontinuous variables, and a chi‐square test or a Fisher's test for categorical variables. Spearman's rank correlation and multiple regression analysis were used to test the associations of human milk vitamins with infant growth indexes. A binomial logistic regression was performed to examine the associations between human milk vitamins and breast milk jaundice and adjusted for related variables. *P* values (two‐tailed) less than 0.05 were considered statistically significant.

## 3 Results

### 3.1 Milk Vitamin Changes During Lactation and Correlation With Maternal Status

**FIGURE 1:**
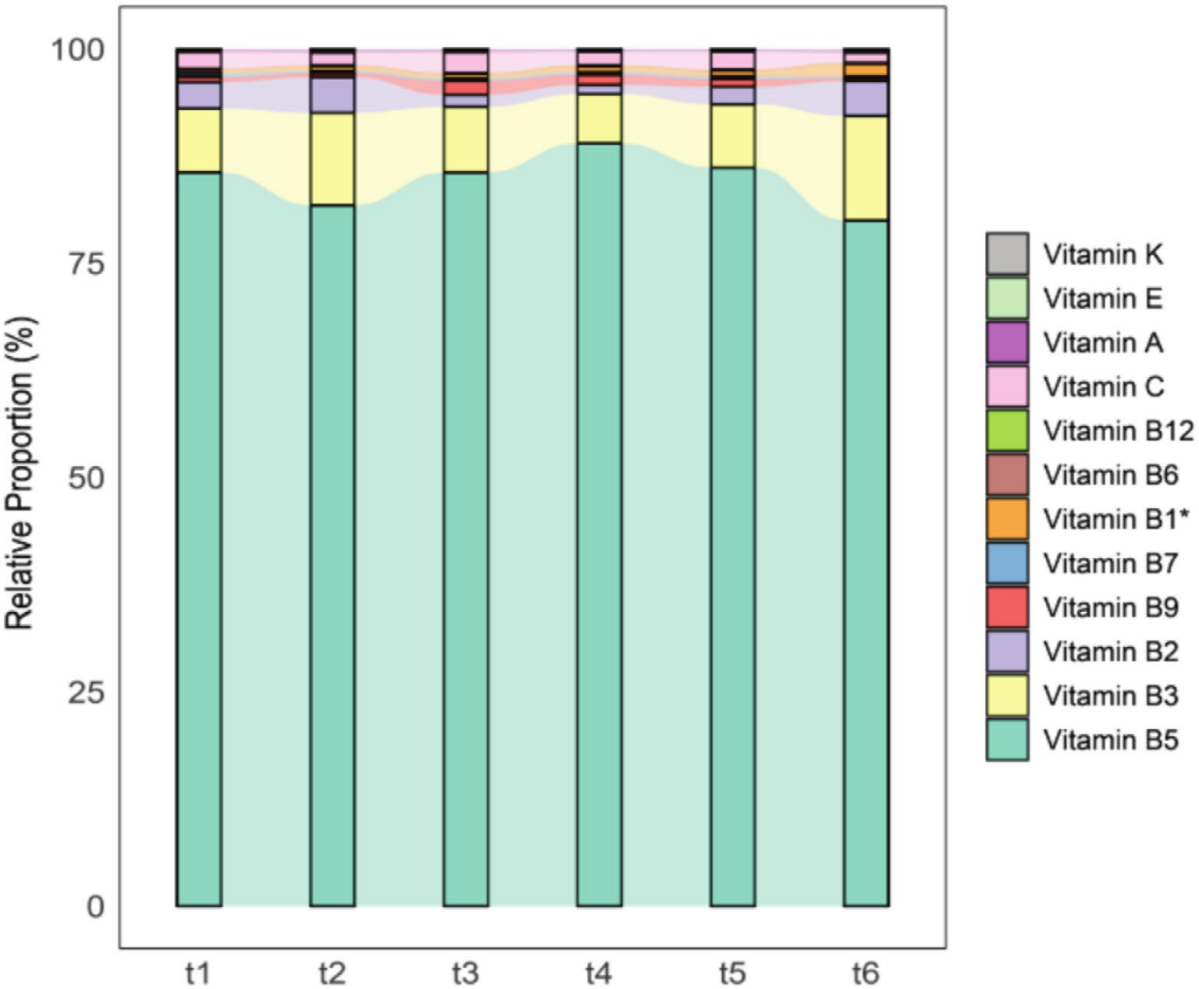
The variational proportion of vitamins in human milk during different lactation stages. T1: 30–44; t2: 45–59; t3: 60–74; t4: 75–89; t5: 90–104; t6: 105–119 days *postpartum*. *The proportion of vitamin B1 results varied with the timing of specimen collection (*p* = 0.01) and was compared by Krustal‐Kallis H.

The vitamins were present in human milk from nursing women with varying concentrations at different stages of lactation. The mean concentrations and proportions of vitamins in human milk from 1 to 3 months are displayed in Figure 1 and Table S1. The content of vitamin B1 was significantly higher at 105 to 119 days postpartum than it was at 30 to 44 days after delivery (adj–*p* = 0.007, Table S1), whereas there were no differences between groups in the other vitamins (*p* > 0.05, Figure 1, Table S1).

This work assessed maternal status in relation to changes in dynamic concentrations of vitamins in human milk through a cohort of 46 mother‐infant pairs. The correlation between maternal factors and the content changes in milk vitamins is summarized in Table S2. The dietary intakes of lactating women conformed to the standards of the Chinese Food Guide Pagoda and Dietary Guidelines for Residents (2016) set by the Chinese Nutrition Society (Wang et al. 2016), which included five categories of foods: grains and potatoes, fruits and vegetables, nuts and seeds, soybean products and oils, and animal‐based foods (meat, poultry, and eggs), respectively. The lactating women with the highest intake of all five categories of foods secreted higher levels of vitamin B1 in human milk than those mothers without consuming soybeans (adj–*p* = 0.01, Table S2). Meanwhile, no significant differences in maternal age, mode of delivery, the grades of pre‐BMI, or parity between groups were observed in our research (*p* > 0.05, Table S2).

### 3.2 Interactions Between Human Milk Vitamin Concentrations and Infant Growth Indexes

**FIGURE 2:**
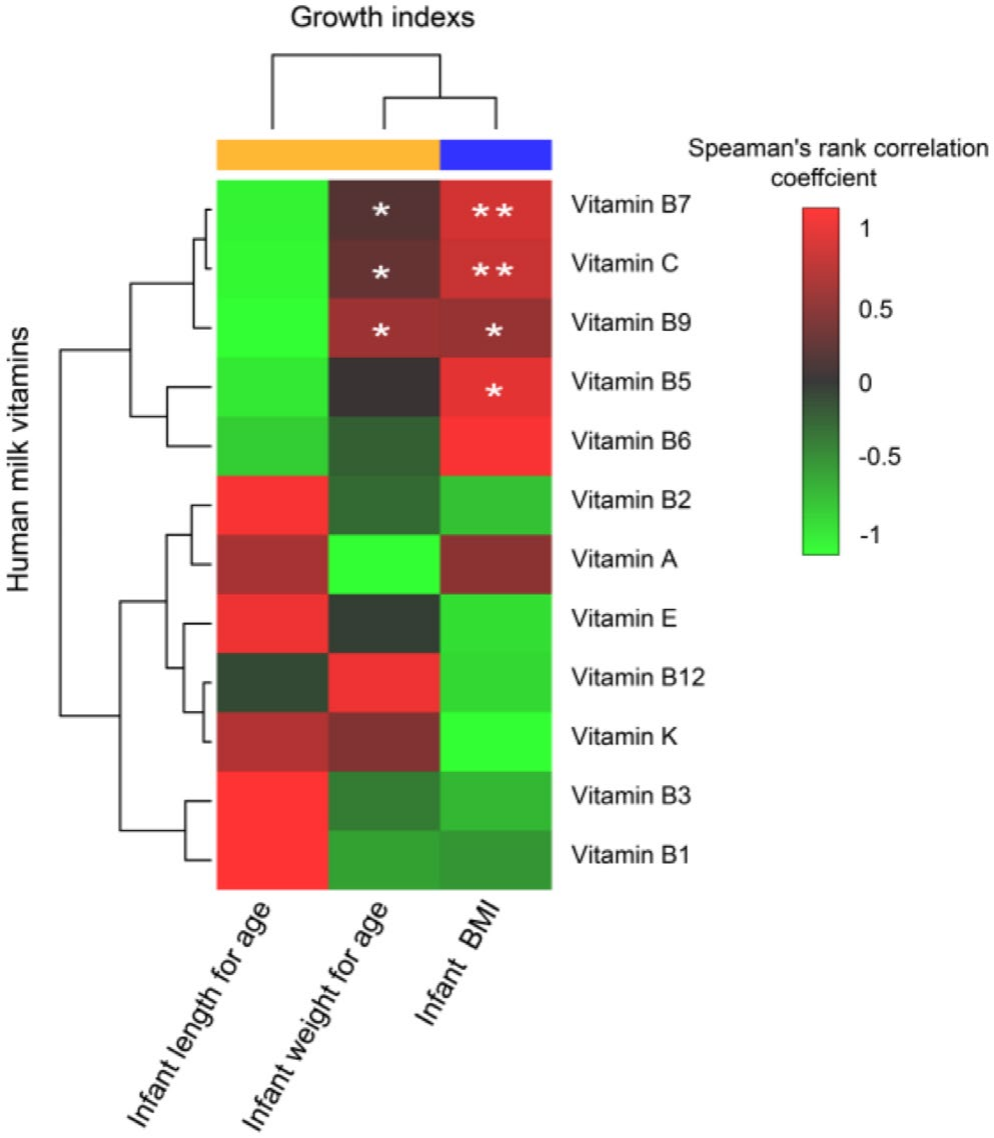
Spearman correlation of human milk vitamins and infant growth indexes. *0.01 ≤ *p* < 0.05, **0.01 < *p* < 0.001.

The correlation between human milk concentrations and infant growth indexes was revealed as shown in Figure 2 and Table S2. A positive correlation was observed between infant weight‐for‐age and vitamins B7, B9, and C, respectively (|*r*| > 0.3, *p* < 0.05). The infant BMI was positively correlated with the concentrations of vitamins B5, B7, B9, and C, respectively, in human milk (|*r*| > 0.3, *p* < 0.05).

To explore the causation between variable concentrations of vitamins in human milk and infant growth factors, the infant growth factors were used as regression dependent variables, and statistically significant vitamins were introduced as regression independent variables according to the correlation analysis. In the final linear regression model, vitamins C and B7 were correlated independently with infant BMI (*p* < 0.05, Table 1). The multiple linear regression analysis results yielded the following formulae: Model 1 (the regression equation of the infant weight‐for‐age was *y* = 30.57 + 0.05 vitamin C, the adjusted *R* ^*2*^ = 0.1) and Model 2 (the regression equation of the infant BMI was *y* = 0.04 + 0.00015 vitamin C + 0.00048 vitamin B7, the adjusted *R* ^*2*^ = 0.21), as shown in Table 1.

**TABLE 1:** Independent correlates of human milk vitamins in infant growth factors among the study subjects in the multivariate analysis using a linear regression model.

Variables	Non‐standardized coefficients		Standardized coefficients	*t*	*p*	VIF	*R* ^2^	D‐W
	Beta (β)	Standard (SE)	Beta (β)					
Model 1^a^								
Constant	30.57	2.05		14.94	< 0.001	1	0.12	2.64
Vitamin C	0.05	0.02		2.4	0.02			
Model 2^b^								
Constant	0.04	0.01		5.74	< 0.001	1.06	0.25	1.95
Vitamin C	0.00015	0.00006	0.35	2.56	0.01			
Vitamin B7	0.00048	0.00023	0.28	2.06	0.04			

*Note:* BMI, body mass index; *R* ^*2*^ for model 1: 0.12, *R* ^*2*^ (adjusted): 0.1; *R* ^*2*^ for model 2: 0.25, *R* ^*2*^ (adjusted): 0.21; Unstandardized and standardized coefficients were determined by multiple linear stepwise regression analysis.

^a^ Model 1: Human milk vitamins B7, B9, and C in relation to the weight‐for‐age in infants aged 1 to 3 months were included as the independent variables.

^b^ Model 2: Human milk vitamins B5, B7, B9, and C in breast milk in relation to infant BMI were included as the independent variables.

### 3.3 Selection of Potential Risk Factors of Vitamins in Human Milk Relating to Breast Milk Jaundice

The contents of vitamin B1 in human milk showed an association with BMJ infants during lactation in single‐factor analysis (*p* = 0.02, Table 2).

**TABLE 2:** Descriptive characteristics of the study subjects and univariate analysis for breast milk jaundice.

	Without event (*n* = 34, 73.91%)	With event (*n* = 12, 26.09%)	*p*
Time, days, *n* (%)^a^			
30–44	7 (20.59)	7 (58.33)	0.07
45–59	8 (23.53)	3 (25)	
60–74	3 (8.82)	2 (16.67)	
75–89	5 (14.71)	0	
90–104	3 (8.82)	0	
105–119	8 (23.53)	0	
Sex, *n* (%)^b^			
Male	22 (64.71)	6 (50)	0.58
Female	12 (35.29)	6 (50)	
Vitamin concentrations (ng/ml), median (IQR)^c^			
Vitamin A	12.32 (9, 18.48)	16.51 (14.03, 22.08)	0.09
Vitamin E	1.9485 (1.46, 3.06)	2.84 (1.91, 3.78)	0.15
Vitamin K	0.3 (0.13, 0.57)	0.56 (0.27, 1.46)	0.08
Vitamin B1	29.3 (20.86, 42.15)	19.91 (15.44, 27.37)	0.02
Vitamin B12	0.32 (0.1, 1.21)	0.32 (0.11, 1.52)	0.66
Vitamin B2	53.31 (21.25, 122.5)	74.63 (35.3, 80.69)	0.75
Vitamin B3	334.21 (215.49, 516.21)	391.37 (313.35, 596.68)	0.11
Vitamin B5	3686.85 (2971.32, 4609.12)	3096.13 (2715.45, 6040.28)	0.73
Vitamin B6	3.52 (2.19, 5.06)	3.02 (2.58, 5.95)	0.53
Vitamin B7	11.69 (6.83, 18.1)	10.69 (8.56, 24.98)	0.62
Vitamin B9	16.54 (7.1, 29.07)	29.78 (9.72, 33.7)	0.24
Vitamin C	64.94 (39.58, 101.8)	85.28 (45.47, 229.48)	0.17

*Note:* Characteristics data of without and with breast milk jaundice were reported as median (interquartile range) for normal variables and number (percentage) for categorical variables.

^a^ Compared by Fisher's precision probability test.

^b^ Compared by Yates's correction for the continuity test.

^c^ Compared by the Mann–Whitney *U* test.

Considering that there was limited power to detect interactions, variables whose statistical significance was lower than 0.1 in the univariate analysis were included in the final logistic regression analysis, as previously recommended (Altman and Bland 2003). After adjustment for specific confounders (age, vitamin A, and vitamin B1), we found that the BMJ in infants aged 1 to 3 months was related to an increase in human milk vitamin K concentrations, with the odds ratio of 13.93 (95% confidence interval, 0.93 to 208.48; *p* = 0.05 by logistic regression, Table 3). In view of Model 3, the validity and advancement of the diagnostic model were verified by comparison and analysis of the results (AUC = 0.849, 95% confidence interval, 0.733 to 0.966; *p* = 0.00036, Figure S1).

**TABLE 3:** Odds ratios and 95% confidence intervals for the associations between the related variables and breast milk jaundice in the logistic regression analysis.

Variables	OR (95% CI)	*p*
Model 1		
Vitamin K	3.98 (1.09, 14.49)	0.03
Model 2		
Vitamin K	11.47 (1.02, 128.41)	0.04
Model 3		
Vitamin K	13.93 (0.93, 208.48)	0.05

*Note:* Binary logistic regression analysis (in fully adjusted and stepwise backward methods) was performed to specify the association between changes in milk vitamin content and breast milk jaundice in infants aged 1 to 3 months. Model 1: unadjusted; Model 2: adjusted for sample collection times only; Model 3: vitamins A and B1 were adjusted on the basis of Model 2.

Abbreviation: OR, odds ratio.

## 4 Discussion

As a result, we found that the concentrations of vitamins C and B7 in human milk were discovered to be associated with higher body mass index in infants aged 1 to 3 months. A positive correlation was presented between infant weight‐for‐age and the concentrations of vitamin C in human milk. The concentrations of vitamin B1 in human milk exhibited differences over lactation duration, which may be affected by mothers who consumed soybeans. Potential human milk vitamin K diagnostic markers may be identified as distinguishing the BMJ in infants.

This study had a higher proportion of vitamin B5, followed by vitamin B3 and vitamin B9, which is consistent with the findings from Redeuil, K., and colleagues. They further reported that vitamin B1 concentrations in human milk reached a peak at 8 weeks postpartum and fell thereafter (Redeuil et al. 2021). In contrast, our results show only part of the vitamin B1 concentrations within these trends. A report regarding content changes in vitamin B1 with time within feedings is in keeping with our findings (Han et al. 2023). The potential confounding factors, such as variable times of sampling collection (Hampel et al. 2017), analytical techniques (Hampel and Allen 2016; Hampel et al. 2022), regional factors (Ren et al. 2015), and especially the diet of the study population (Ortega et al. 2004), are taken into consideration to influence the variances in milk vitamin concentrations among women. Mammary glands lack the ability to synthesize water‐soluble vitamins, the main source of which derives from the maternal plasma (Yaman et al. 2021) rather than supplementation with micronutrients (Allen et al. 2015). The composition of water‐soluble vitamins in human milk is influenced by mothers' dietary patterns during the lactation period (Qiao et al. 2022; Xue et al. 2017). Contrarily, a systematic review assessed and confirmed that the evidence is insufficient (Scinto‐Madonich et al. 2021). In fact, our study demonstrated that human milk vitamin B1 generally showed upward trends over time. Whereas compared to postpartum women within 105–119 days, the maternal deficiency of soy appears to be higher in populations postpartum within 30 to 44 days (Figure S2). In addition, there is a high prevalence of vitamin B1 deficiency in the many populations that consume low amounts of soybeans. In this regard, vitamin B1 widely exists in soy products, and studies have agreed that diets supplemented with soybeans have been associated with an increase in the vitamin B1 concentrations in human blood (Fattal‐Valevski et al. 2005; Lebiedzińska and Szefer 2006). Consequently, it can be assumed that vitamin B1 concentrations in human milk are likely dependent on the maternal intake of soybean products in our results.

In this research, mothers' milk with higher levels of vitamins B7 and C was better present in their infants' body indexes. Generally, maternal vitamin status is important to sufficiently provide infants with nutrition. Biotin decreased throughout the lactation period (Q. Ren et al. 2024) and is a component of carboxylase enzymes that are vital for amino acid metabolism (León‐Del‐Río 2019), gluconeogenesis, fatty acid biosynthesis, and odd‐chain fatty acid catabolism (Allen and Hampel 2019). Vitamin C is involved in the metabolism of amino acids and folic acid (Golding 2018; Na et al. 2006), and it is also important for the utilization of carbohydrates (Pham et al. 2021), fat (Garcia‐Diaz et al. 2014), and protein (Na et al. 2006). Further evidence comes from a study with Chinese primary school students where lower dietary sources of vitamin C did not meet physical demands during this critical phase of development (Liu et al. 2019). The precise levels of vitamins in human milk are almost unknown, as are the effects of vitamin concentrations on infant growth and development. Currently, our preliminary observational data indicate the levels of vitamins B7 and C in human milk and their positive relationship to infant body growth indicators. The remaining challenge is to document if there is a causal link between marginal maternal vitamin B7 and C status, milk vitamin B7 and C, and infant development.

Following its transport via the bloodstream to the liver, unconjugated bilirubin is actively taken up into hepatocytes by specific transporter proteins located on the sinusoidal membrane. Within the hepatocyte, bilirubin binds to cytosolic ligandins, primarily Y‐protein (glutathione S‐transferase B, GST‐B) and Z‐protein (fatty acid‐binding protein, FABP). This binding prevents bilirubin reflux into the circulation and facilitates its transport to the endoplasmic reticulum for subsequent metabolic conjugation (Arias 2022). In infants with breast milk jaundice, human breast milk components have been shown to inhibit Z protein‐bromosulfophthalein binding within hepatocytes (Foliot et al. 1976). This inhibition impairs the hepatocellular uptake capacity for bilirubin. Notably, Z protein is a vitamin K‐dependent glycoprotein isolated from humans (Ichinose et al. 1990; Sejima et al. 1990).

Despite variations in maternal diet and geography, human milk universally fails to deliver sufficient vitamin K (< 5 μg/L) to meet the 2 to 5 μg/day requirement for full‐term infants under 6 months of age (Hand et al. 2022; Nations 2005). The Adequate Intake (AI) for vitamin K for Chinese infants between 0 and 5 months is 3.49 μg/d (2.18 μg/d for vitamin K1 and 1.06 μg/d for vitamin K2) (Wang et al. 2024). Vitamin K in human milk is insufficient to meet recommended intakes for infants aged less than 6 months (Canfield et al. 1991).

Fat‐soluble vitamins can be absorbed from the diet through the small intestine along with dietary fat and stored in the liver and fat tissue until required (Alamri 2018). For infants who have not taken in complementary food, the two sources of vitamin K are human milk and their own endogenous gut bacteria (Ellis et al. 2022). In contrast to formula‐fed infants, the intestinal microbiome of exclusively breastfed infants exhibits negligible vitamin K2 production (Greer 2004). Vitamin K in human milk is typically provided directly to infants whose nutrition is the norm (Canfield and Hopkinson 1989). In the case of a vitamin polarity, the fat‐soluble vitamin K requires carrier proteins or lipoprotein vesicles for transport through the body (Shearer 1992). Although the effect of vitamin K on BMJ is unknown, it can compete with bilirubin for binding sites on albumin, leading to an increase in free bilirubin. A consideration is that, due to immature liver function and potential damage to the liver by vitamin K, infants have poor handling of bilirubin (Asteriadou‐Samartzis and Leikin 1958). Vitamin K may cause prolonged jaundice via hepatic uptake, hepatic excretion, and conjugation, thereby raising the need for further studies to investigate a possible mechanism contributing to the occurrence of BMJ. Despite the significant yet small differences found in vitamin B1 in human milk between the two groups in the univariate analysis, they were unaffected based on the logistic regression analysis. One possible explanation was that vitamin B1 interacted with collection times.

The limitations of this study are as follows: The observed data is poor due to the small number of participants. Larger variations were present, which could reflect the differences in maternal dietary intake assessment methods and maternal nutritional status between studies, making it even more difficult to interpret the results. Moreover, the external validity of our findings needs to be interpreted with caution owing to the fact that these data are part of a monocentric and exploratory study. Furthermore, this study was a cross‐sectional study, so the conclusions drawn could not be further causally inferred. In the future, we will expand the size of the mother‐infant cohort and conduct in‐depth longitudinal research, laying the groundwork for maternal interventions to optimize vitamin composition in human milk and their implications for later offspring outcomes.

## 5 Conclusion

In conclusion, the variations of water‐soluble vitamin B1 in human milk may present a main association with maternal dietary intakes of soybeans. Adequate contents of vitamins B7 and C in the human milk supply for an infant's body growth from 1 to 3 months. In addition, our results implied that the BMJ in infants was related to an increase in human milk vitamin K. Our findings suggest that increased maternal soybean product consumption at 1 month postpartum may enhance vitamin B1 concentrations in human milk. Furthermore, maintaining adequate levels of vitamins B7 and C in human milk is essential to ensure infant growth. In view of the results of this study, further studies are warranted for the BMJ infant role of vitamin K in mature milk.

## Author Contributions

**Yuanyuan Zhang:** conceptualization (equal), data curation (lead), formal analysis (lead), investigation (equal), methodology (lead), resources (equal), visualization (lead), writing – original draft (lead). **Xuerong Zhang:** data curation (equal), methodology (equal). **Zhenrong Xie:** supervision (equal), validation (equal). **Jingjing Xiong:** resources (equal), supervision (equal). **Meng Li:** writing – review and editing (equal). **Zhanhua Li:** data curation (equal), methodology (equal), visualization (equal). **Yongkun Huang:** conceptualization (equal), methodology (equal), project administration (lead), validation (equal).

## Ethics Statement

The study protocol with all procedures involving human subjects was approved by the ethics committee of the First Affiliated Hospital of Kunming Medical University (Protocol # (2020) L–23). The trial was performed in accordance with the ethical standards laid down in the Declaration of Helsinki and its later amendments. Written informed consent was obtained from the participants' legal guardian or next of kin. Participation was voluntary, and participants were free to withdraw from the study at any stage.

## Conflicts of Interest

The authors declare no conflicts of interest.

## Supporting information


**Table S1:** The changes of human breast milk vitamin contents during different lactation periods.
**Table S2:** Baseline characteristics of mother participants according to human breast milk vitamin concentrations.
**Table S3:** The correlation between infant growth indexes and different human milk vitamins.
**Figure S1:** ROC analysis for the predictive model of infants with breast milk jaundice and human milk vitamin K.
**Figure S2:** The different dietary intake proportions of mothers in two stages of postpartum.

## Data Availability

All data analyzed during this study are included in this published article (and its Supporting Information). The raw data supporting the conclusions of this article will be made available by the authors on request.
