# Supplementary material for: From Mother to Baby: The Role of Human Milk Vitamins in Infant Body Development and Breast Milk Jaundice—An Observational Study
Source: Food Sci Nutr. 2025 Sep 15;13(9):e70922. doi: 10.1002/fsn3.70922 (PMC12436415; doi:10.1002/fsn3.70922)
Supplement: Supplementary file 1 — Table S1: The changes of human breast milk vitamin contents during different lactation periods. Table S2: Baseline characteristics of mother participants according to human breast milk vitamin concentrations. Table S3: The correlation between infant growth indexes and different human milk vitamins. Figure S1: ROC analysis for the predictive model of infants with breast milk jaundice and human milk vitamin K. Figure S2: The different dietary intake proportions of mothers in two stages of postpartum. [file FSN3-13-e70922-s001.docx]

**Supporting Information**

**From Mother to Baby: The Role of Human Milk Vitamins in Infant Body Development and Breast Milk Jaundice****—An Observational Study**

**Supplementary Information**

**Supplementary Method**

**Files in this data supplement**

Supplementary Table S1

Supplementary Table S2

Supplementary Table S3

Supplementary Figure S1

Supplementary Figure S2

**Supplementary Method**

i) Product information of isotopic-labeled internal standards:

| VB1 | B1-IS |
| --- | --- |
| VB2 | B2-IS |
| VB3 | B3-IS |
| VB5 | B5-IS |
| VB6 | B6-IS |
| VB7 | B7-IS |
| VB9 | B9-IS |
| VB12 | B12-IS |
| VA | VA-IS |
| VC | VC-IS |
| VK | VK-IS |
| VE | VE-IS |

ii) Product information of UPLC: A Shimadzu LC-AD ultrafast liquid chromatography system (Shimadzu, Tokyo, Japan) and an API 4500 triple quadrupole mass spectrometer (SCIEX, Framingham, MA, USA).

iii) Solvent composition used for the analysis:

| Water-Soluble Vitamins | |
| --- | --- |
| Product Component | Primary Composition |
| Extraction Solution | Acetonitrile |
| Mobile Phase A | Aqueous solution of formic acid and ammonium formate |
| Mobile Phase B | Methanolic solution of formic acid and ammonium formate |
| Stabilizer | Aqueous solution of citric acid and 2-hydroxyethanol |
| Calibrator | VB1, VB2, VB3, VB5, VB6, VB7, VB9, VB12, VC |
| Quality Control I | VB1, VB2, VB3, VB5, VB6, VB7, VB9, VB12, VC |
| Quality Control II | VB1, VB2, VB3, VB5, VB6, VB7, VB9, VB12, VC |
| Internal Standard | VB1 IS, VB2 IS, VB3 IS, VB5 IS, VB6 IS, VB7 IS, VB9 IS, VC IS |
| Calibrator Diluent | Contains BSA |

| Lipid-Soluble Vitamins | |
| --- | --- |
| Product Component | Primary Composition |
| Extraction Solution A | Acetonitrile |
| Extraction Solution C | n-hexane |
| Mobile Phase A | Formic acid and ammonium |
| Reconstitution Solution | Formic acid and ammonium formate in methanol: water solution |
| Mobile Phase B | Formic acid and ammonium formate in methanol solution |
| Preservative Solution | Citric acid and 2-hydroxyethanol in aqueous solution |
| Calibrator | VA, VE, VK |
| Quality Control I | VA, VE, VK |
| Quality Control II | VA, VE, VK |
| Internal Standard | VA IS, VE IS, VK IS |
| Calibrator Diluent | Contains BSA |

iv) Quantification transitions and collision energy:

| Result of product ion scanning of breast milk vitamins using an AB 4500MD system. | | | |
| --- | --- | --- | --- |
| Compound | m/z | | Collision Energy (eV) |
|  | Precursor Ion | Product Ion |  |
| Vitamin A | 269.4 | 81.4 | 27 |
| Vitamin E | 431.1 | 165.5 | 27 |
| Vitamin K | 451.3 | 187.3 | 34 |
| Vitamin B1 | 265.3 | 144 | 21 |
| Vitamin B2 | 37 | 17 | 31 |
| Vitamin B3 | 12 | 78 | 28 |
| Vitamin B5 | 220 | 72 | 21 |
| Vitamin B6 | 18 | 14 | 29 |
| Vitamin B7 | 24 | 91 | 23 |
| Vitamin B9 | 460 | 180 | 25 |
| Vitamin B12 | 67 | 13 | 55 |
| Vitamin C | 17 | 95 | 14 |

**Supplementary Tables**

| **TABLE S1.** The changes of human breast milk vitamin contents during different lactation periods. | | | | | | | |
| --- | --- | --- | --- | --- | --- | --- | --- |
| Vitamin contents (ng/ml), mean ± standard deviation (SD) or median (IQR) | Subject collection times (n) | | | | | | P  value |
|  | 30–44 days (n = 14) | 45–59 days (n = 11) | 60–74 days (n = 5) | 75–89 days (n = 5) | 90–104 days (n = 3) | 105–119 days (n = 8) |  |
| Vitamin A | 16.46 (9,  22.99) | 16.74 (13.91, 19.89) | 11.65 (9.57, 20.94) | 10.45 (9,  21.66) | 11.33 ± 1.24 | 11.87 (9.03, 17.64) | 0.5 |
| Vitamin E | 1.92 (1.06, 3.31) | 2.20 (1.70, 4.20) | 1.91 (1.16, 3.72) | 2.6 (0.93, 3.06) | 2.24 ± 0.40 | 2.92 (1.44, 5.42) | 0.86 |
| Vitamin K | 0.47 (0.21, 1.18) | 0.53 (0.30, 0.74) | 0.20 (0.08, 0.35) | 0.23 (0.10, 0.79) | 0.19 ± 0.10 | 0.60 (0.20, 1.35) | 0.24 |
| Vitamin B1 | 20.85 (15.78, 27.37)^a^ | 22.14 (17.93, 42.82) | 18.20 (11.62, 36.70) | 28.44 (22.52, 57.28) | 31.38 ± 6.45 | 41.51 (38.18, 92.65) | **0.01** |
| Vitamin B12 | 0.40 (0.10, 0.98) | 0.14 (0.10, 0.41) | 0.32 (0.12, 2.26) | 1.16 (0.72, 3.96) | 0.10 ± 0.01 | 0.44 (0.10, 1.93) | 0.1 |
| Vitamin B2 | 68.90 (41.05, 105.84) | 24.65 (9.57, 76.27) | 81.34 (41.10, 82.43) | 49.24 (25.99, 95.06) | 94.07 ± 36.98 | 87.17 (22221.85) | 0.41 |
| Vitamin B3 | 349.02 (301.17,  619.80) | 342.70 (196.91,  464.29) | 342.44 (244.78,  461.48) | 355.72 (156.31,  445.47) | 341.91 ± 99.77 | 469.61 (256.71,  627.01) | 0.87 |
| Vitamin B5 | 4049.70 (3339.46, 5872.74) | 3502.53 (2588.10, 4594.78) | 3346.45 (2423.84, 5710.72) | 3605.16 (2418.28, 7874.90) | 3979.27 ± 300.47 | 3011.71 (1910.61, 4210.28) | 0.45 |
| Vitamin B6 | 4.21 (2.54, 6.59) | 2.46 (1.89, 3.45) | 3.25 (2.39, 5.50) | 3.88 (2.42, 5.43) | 4.37 ± 0.44 | 3.43 (2.21, 5.90) | 0.49 |
| Vitamin B7 | 15.52 (10.09, 34.88) | 9.81 (7.11, 12.26) | 11.82 (6.85, 20.93) | 7.23 (2.92, 36.10) | 14.12 ± 2.60 | 6.80 (4.69, 11.70) | 0.07 |
| Vitamin B9 | 24.56 (4.59, 33.08) | 26.65 (8.15, 32.09) | 27.31 (11.33, 162.39) | 17.74 (11.17, 141.38) | 43.13 ± 14.89 | 14.31 (7.95, 22.79) | 0.66 |
| Vitamin C | 85.28 (39.08, 170.79) | 64.15 (47.94, 77.59) | 121.65 (63.75, 151.13) | 57.25 (43.22, 141.05) | 96.48 ± 27.77 | 38.12 (25.98, 69.91) | 0.25 |

*Note:* Normal human milk vitamins were expressed as means ± standard deviation (SD); nonnormal human milk vitamins were expressed as median (interquartile range) and were compared by Krustal-Kallis H. ^a^ Compared human milk vitamin B1 in 30–44 days postpartum (20.84, 15.78 to 27.37) to 105–119 days postpartum (41.51, 38.18 to 92.65) by Dunn's post hoc test for multiple comparison (*z* = -20.91, adj-*p* value = 0.007).

| **TABLE S2.** Baseline characteristics of mother participants according to human breast milk vitamin concentrations. | | | | | | | | | | | | |
| --- | --- | --- | --- | --- | --- | --- | --- | --- | --- | --- | --- | --- |
| Vitamin concentrations (ng/ml), median (IQR) | Mother's age, No (%)^a^ | | Gestational weight gain, No (%)^b, ([Rasmussen and Yaktine 2009](#_ENREF_1))^ | | | Delivery mode, No (%)^a^ | | Parity, No (%)^a^ | | Diet, No (%)^b^ | | |
|  | <35 years, 38 (82.61) | ≥35 years, 8 (17.39) | Below guidelines, 22 (47.83) | Follow guidelines, 14 (30.43) | Above guidelines, 10 (21.74) | Vaginal,  32 (69.57) | Cesarean, 14 (30.43) | Primiparous, 29 (63.04) | Multiparous, 17 (36.96) | Intake food with five categories,  27 (58.70) | Grain and potato deficiency,  4 (8.70) | Soybean deficiency,  15 (32.6) |
| Vitamin A | 14.15 (9, 19.018) | 15.73 (12.04, 18.92) | 15.49 (9, 19.02) | 12.5 (9, 20) | 14.57 (9.08, 20.3) | 15.49 (10.22, 19.68) | 12.67 (9, 17.12) | 13.91 (9.06, 18.35) | 14.75 (9, 20.66) | 10.95 (9, 18.37) | 20.31 (11.33, 38.43) | 15.59 (11.79, 24.98) |
| Vitamin E | 1.95 (1.53, 3.16) | 3.07 (1.92, 5.86) | 2.06 (1.46, 3.28) | 2.63 (1.70, 3.29) | 1.88 (1.25, 2.91) | 2.28 (1.65, 3.74) | 1.92 (1.32, 2.8) | 2.4 (1.68, 3.48) | 1.95 (1.42, 2.86) | 2.16 (1.51, 3.23) | 1.75 (0.63, 2.88) | 2.56 (1.91, 3.54) |
| Vitamin K | 0.45 (0.18, 0.81) | 0.26 (0.12, 0.38) | 0.32 (0.2, 0.57) | 0.48 (0.11, 1.2) | 0.39 (0.12, 1.08) | 0.38 (0.2, 0.66) | 0.37 (0.13, 1.16) | 0.31 (0.09, 0.54) | 0.46 (0.2, 1.28) | 0.3 (0.2, 0.65) | 0.94 (0.16, 1.46) | 0.39 (0.09, 0.74) |
| Vitamin B1 | 28.09 (18.9, 42.15) | 23.13 (18.63, 33.09) | 28.19 (19.46, 39.18) | 24.66 (16.79, 41.12) | 26.62 (18.97, 43.65) | 28.25 (17.99, 38.61) | 24.45 (20.65, 49.66) | 24.74 (18.71, 35.14) | 33.69 (18.53, 46.96) | 32.97 (20.88, 48.79) | 25.67 (15.95, 37.61) | 19.88 (14.88, 28.25)^c^ |
| Vitamin B12 | 0.32 (0.1, 1.03475) | 0.99 (0.1, 2.68) | 0.32 (0.1, 1.74) | 0.27 (0.1, 1.1) | 0.24 (0.1, 1.25) | 0.31 (0.1, 0.92) | 0.92 (0.1, 2.35) | 0.41 (0.1, 1.5) | 0.15 (0.1, 0.62) | 0.14 (01, 0.97) | 1.81 (0.46, 5.22) | 0.41 (0.12, 1.62) |
| Vitamin B2 | 50.42 (21.25, 86.22) | 80.04 (66.51, 113.02) | 66.59 (30.41, 86.22) | 60.09 (19.65, 140.75) | 54.04 (31.1895, 85.442) | 55.75 (22.6, 91.33) | 69.37 (34.81, 132.77) | 51.6 (23.28, 89.04) | 74.15 (32.74, 106.73) | 49.24 (24.65, 96.12) | 133.51 (88.62, 528.92) | 56.47 (21.91, 78.73) |
| Vitamin B3 | 342.57 (226.1, 534.05) | 426.76 (278.25, 539.87) | 355.71 (257.98, 475.33) | 324.57 (226.1, 611.26) | 364.36 (253.67, 580.4) | 355.58 (224.9, 531.71) | 349.21 (271.55, 611.26) | 355.72 (212.2, 483.08) | 342.7 (286.71, 585.92) | 355.72 (276.35, 464.29) | 630.02 (235.43, 688.05) | 325.04 (218.78, 539.46) |
| Vitamin B5 | 3617.48 (2817.34, 4572.18) | 4506.42 (2763.90, 6664.68) | 3553.85 (2726.81, 5037.9) | 3444.66 (3020.98, 4431.31) | 4223.31 (2677.31, 6616.6) | 3781.28 (2802.67, 4828.87) | 3458.77 (2781.25, 4687.46) | 3431.61 (2721.13, 4608.41) | 3818.66 (3212.94, 5213.15) | 3629.79 (2926.27, 5788.97) | 3711.91 (2677.38, 4140.33) | 3474.32 (2709.78, 4594.78) |
| Vitamin B6 | 3.3 (2.19, 5.25) | 3.82 (2.9, 5.49) | 3.2 (2.23, 5.71) | 3.99 (2.42, 5.0) | 3.43 (2.08, 5.93) | 3.18 (2.21, 4.98) | 4.28 (2.54, 5.61) | 3.25 (2.46, 5.37) | 3.45 (2.22, 5.38) | 4.05 (2.23, 6.06) | 3.25 (2.49, 5.6) | 2.7 (2.16, 3.66) |
| Vitamin B7 | 11.62 (7.2, 19.11) | 12 (8.31, 43.6) | 10.89 (6.86, 23.31) | 10.85 (8.47, 14.87) | 11.82 (8.23, 19.08) | 10.53 (7.31, 17.671) | 13.48 (8.97, 32.74) | 10.14 (7.39, 17.45) | 13.24 (7.86, 19.48) | 12.06 (8.6, 20.46) | 20.31 (6.27, 49.68) | 9.6 (7.23, 13.87) |
| Vitamin B9 | 20.69 (7.1, 31.18) | 28.69 (7.59, 57.57) | 22.53 (5.85, 32.24) | 22.52 (11.22, 29.28) | 17.88 (4.59, 32.89) | 20.69 (7.37, 31.6) | 21.43 (7.05, 34.84) | 21.36 (8.51, 29.78) | 15.27 (5.36, 38.7) | 20.84 (7.11, 31.84) | 8.78 (5.4, 198.67) | 26.65 (8.15, 34.32) |
| Vitamin C | 67.67 (37.72, 123.73) | 69.53 (40.22, 144.12) | 64.5 (37.28, 132.81) | 73.48 (63.04, 105.88) | 59.37 (39.58, 144.51) | 65.76 (38.37, 121.15) | 73.57 (40.03, 176.71) | 70.03 (48.33, 128.8) | 63.28 (36.84, 117.6) | 65.79 (40.29, 135.96) | 34.19 (23.11, 162.12) | 72.28 (57.25, 121.65) |

*Note:* Categorical variables presented as number (percentages) and continuous data as median (interquartile range) when they followed a nonparametric distribution. ^a^ Evaluated by the Mann-Whitney U test. ^b^ Compared by Krustal-Kallis H. ^c^ Indicated a significant difference among the three groups (*p* < 0.05). The content of vitamin B1 in human milk from nursing women without intake of soybean products was compared to those who eat five category foods by Dunn's post hoc test for multiple comparison (adj-*p* = 0.01).

| **TABLE S3.** The correlation between infant growth indexes and different human milk vitamins. | | | |
| --- | --- | --- | --- |
| Correlation coefficient | A1 | A2 | A3 |
| Vitamin A | 0.06 | 0.11 | 0.11 |
| Vitamin E | -0.03 | 0.1 | -0.14 |
| Vitamin K | -0.02 | -0.01 | -0.08 |
| Vitamin B1 | -0.27 | -0.12 | -0.26 |
| Vitamin B12 | 0.01 | -0.04 | -0.07 |
| Vitamin B2 | 0.07 | 0.2 | 0.03 |
| Vitamin B3 | -0.17 | -0.03 | -0.2 |
| Vitamin B5 | 0.13 | -0.09 | 0.34^*^ |
| Vitamin B6 | -0.05 | -0.13 | 0.14 |
| Vitamin B7 | 0.3^*^ | 0.05 | 0.44^**^ |
| Vitamin B9 | 0.35^*^ | 0.06 | 0.35^*^ |
| Vitamin C | 0.31^*^ | 0.03 | 0.42^**^ |

*Note:* A1, the weight for age in infants; A2, the length for age in infants; A3, infant BMI. Correlation coefficients between the infant growth indexes and different variables were determined by Spearman’s rank correlation. *0.01 ≤ *p* < 0.05; **0.01 < *p* < 0.001.

**Supplementary Figures**

**
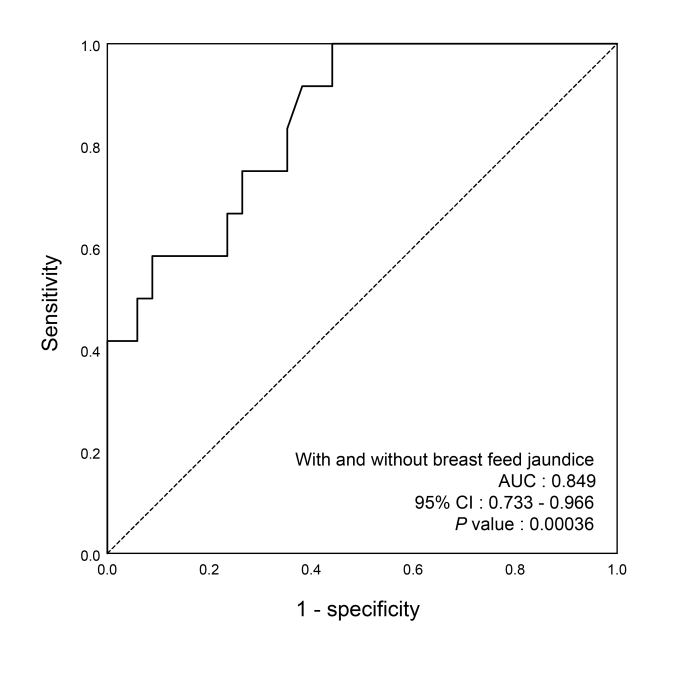
**

**FIGURE S1.**

ROC analysis for the predictive model of infants with breast milk jaundice and human milk vitamin K.


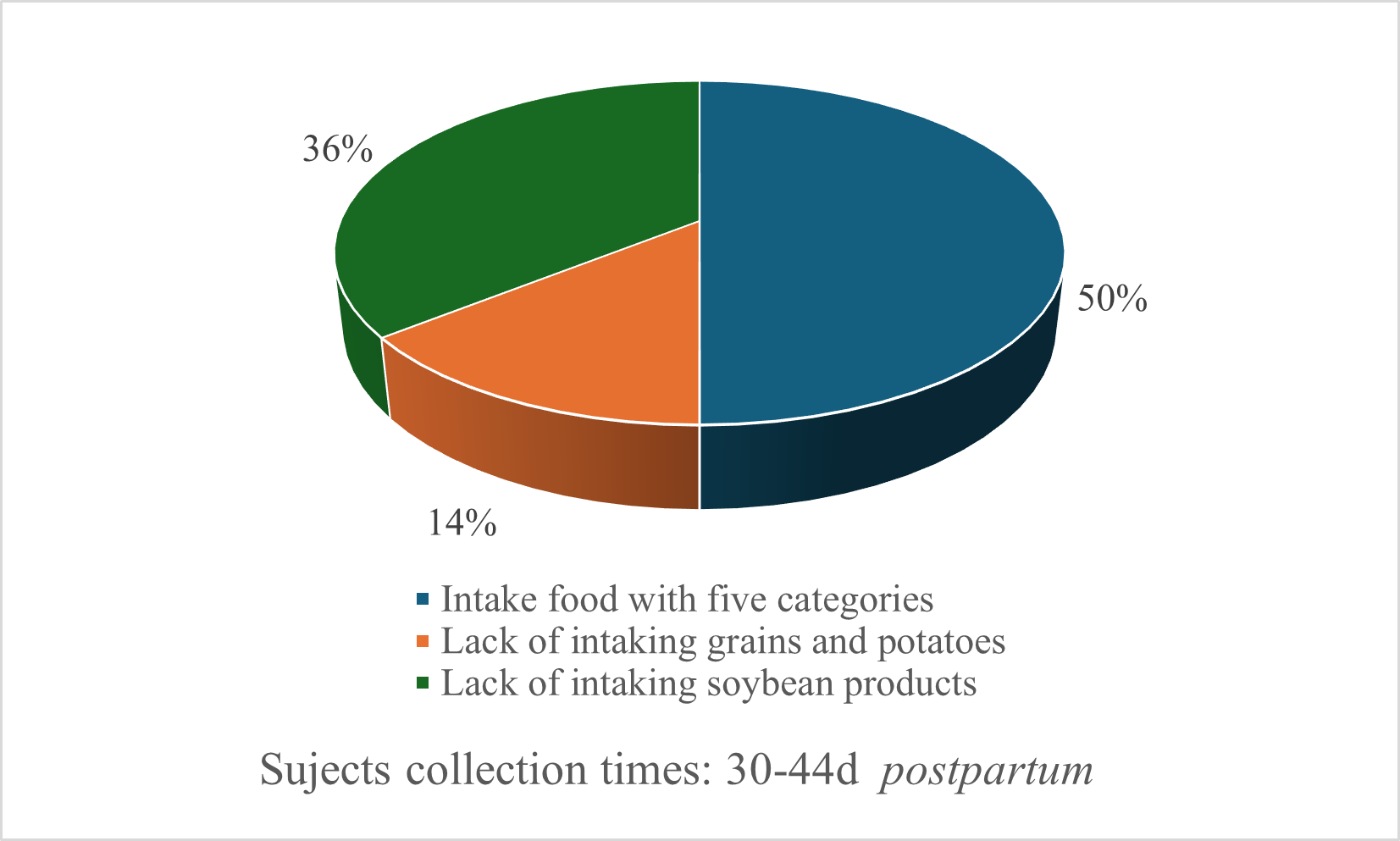

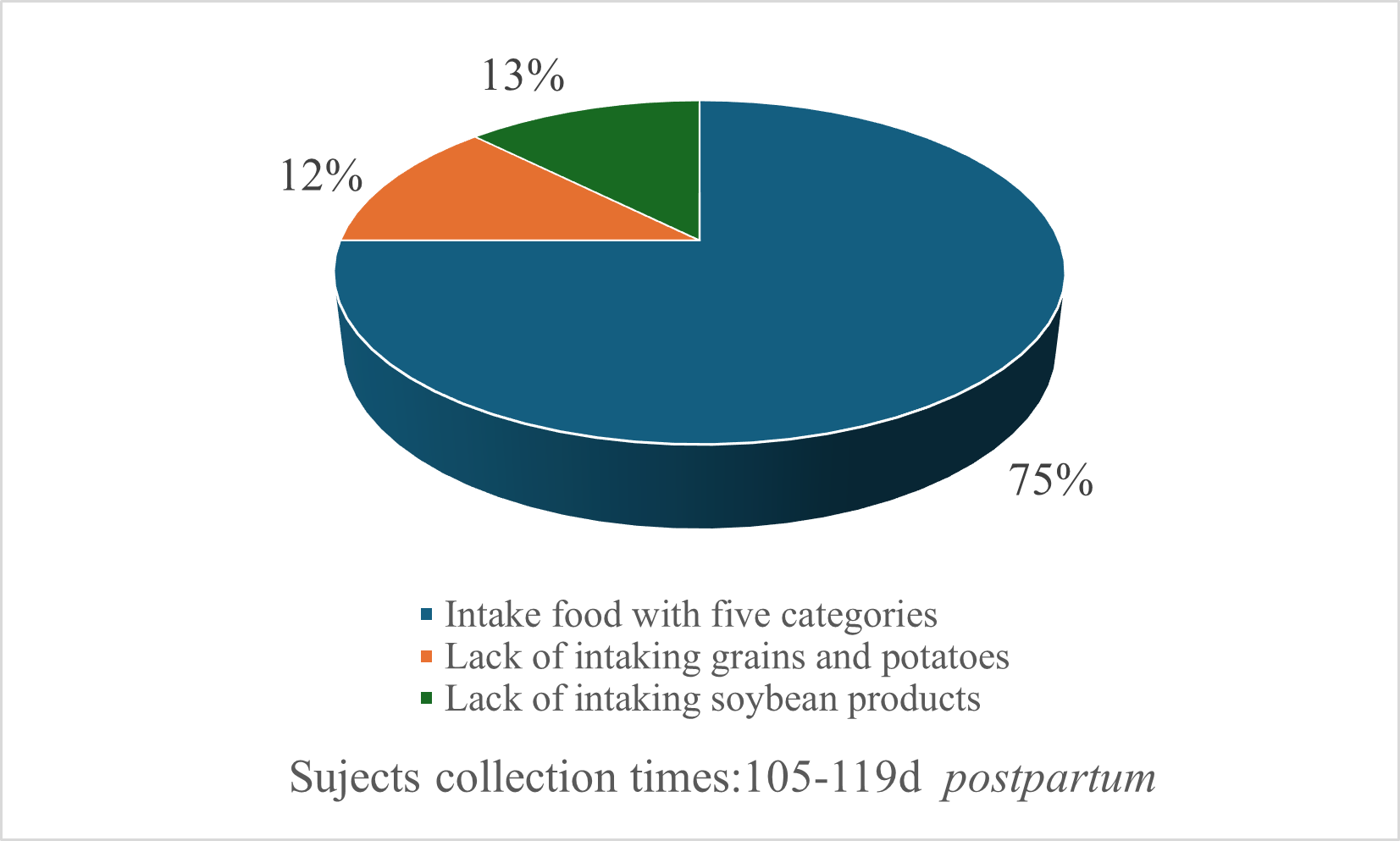


**FIGURE S2.**

The different dietary intake proportions of mothers in two stages of postpartum.

**Reference**

Rasmussen, K. M., and A. L. Yaktine. 2009. *Weight gain during pregnancy: Reexamining the guidelines.*

National Academies Press.
